# Trials within trials of interventions to improve recruitment and retention: the experience of York Trials Unit

**DOI:** 10.1186/1745-6215-16-S2-O9

**Published:** 2015-11-16

**Authors:** David Torgerson, Caroline Fairhurst

**Affiliations:** 1 University of York, York, UK

## Background

Few trials of interventions have been undertaken to improve trial recruitment and retention with two recent Cochrane finding only 57 trials. This could be improved if all of the UK Trials Units agreed to conduct at least two embedded trials per year. At the York Trials Unit (YTU) we have a policy of routinely embedding a recruitment or retention trial in most of our existing studies.

## Method

A review of all YTU ‘trials within trials’.

## Results

Of the 20 trials undertaken 8 were of interventions to enhance recruitment and 12 were of attrition reduction. For the recruitment strategies different information methods (6) were the most common with trial co-ordinator visit and different envelope colours being the other interventions. For the 12 attrition prevention trials 4 were of electronic reminders, 2 of newsletters, 4 were of post-it notes, 1 was of a free pen, and 1 was of offer of trial results. Currently, we have found that electronic reminders and participant newsletters for retention are effective and these are now being routinely included in our trials. Most of our trials were ‘unfunded’ and consequently the interventions tended to be inexpensive. The benefits of doing embedded trials include: improved knowledge and early publications for the trial co-ordinators. Some Chief Investigators can be hostile viewing them as a ‘nuisance’ but with discussion most reasonable investigators can be persuaded.

## Conclusion

If all trials units did two ‘trials within a trial’ per year then the evidence base would be doubled within a year.

